# Reversal of intramyocellular lipid accumulation by lipophagy and a p62-mediated pathway

**DOI:** 10.1038/cddiscovery.2016.61

**Published:** 2016-08-22

**Authors:** T Lam, R Harmancey, H Vasquez, B Gilbert, N Patel, V Hariharan, A Lee, M Covey, H Taegtmeyer

**Affiliations:** 1 Internal Medicine/Cardiology, McGovern Medical School at The University of Texas Health Science Center at Houston, TX, USA; 2 University of Mississippi School of Medicine, Jackson, MS, USA; 3 Davidson College, Davidson, NC, USA; 4 Keck School of Medicine of USC, Los Angeles, CA, USA

## Abstract

We have previously observed the reversal of lipid droplet deposition in skeletal muscle of morbidly obese patients following bariatric surgery. We now investigated whether activation of autophagy is the mechanism underlying this observation. For this purpose, we incubated rat L6 myocytes over a period of 6 days with long-chain fatty acids (an equimolar, 1.0 mM, mixture of oleate and palmitate in the incubation medium). At day 6, the autophagic inhibitor (bafilomycin A1, 200 nM) and the autophagic activator (rapamycin, 1 *μ*M) were added separately or in combination for 48 h. Intracellular triglyceride (TG) accumulation was visualized and quantified colorimetrically. Protein markers of autophagic flux (LC3 and p62) and cell death (caspase-3 cleavage) were measured by immunoblotting. Inhibition of autophagy by bafilomycin increased TG accumulation and also increased lipid-mediated cell death. Conversely, activation of autophagy by rapamycin reduced both intracellular lipid accumulation and cell death. Unexpectedly, treatment with both drugs added simultaneously resulted in decreased lipid accumulation. In this treatment group, immunoblotting revealed p62 degradation (autophagic flux), immunofluorescence revealed the colocalization of p62 with lipid droplets, and co-immunoprecipitation confirmed the interaction of p62 with ADRP (adipose differentiation-related protein), a lipid droplet membrane protein. Thus the association of p62 with lipid droplet turnover suggests a novel pathway for the breakdown of lipid droplets in muscle cells. In addition, treatment with rapamycin and bafilomycin together also suggested the export of TG into the extracellular space. We conclude that lipophagy promotes the clearance of lipids from myocytes and switches to an alternative, p62-mediated, lysosomal-independent pathway in the context of chronic lipid overload (*P<0.05, **P<0.01, ***P<0.001, ****P<0.0001).

## Introduction

The accumulation of fatty acid metabolites in non-adipose tissues is a well-known phenomenon,^1^ first described as ‘fatty atrophy’, or ‘metamorphosis’, by Virchow.^2^ Today, we know that the intracellular accumulation of neutral TG in these relatively innocuous lipid droplets is accompanied by a more deleterious accumulation of ceramides, diacylglycerols, and other intermediary metabolites of long-chain fatty acid metabolism.^1,3,4^ The consequences eventually mediate cell death, as observed in pancreatic *β*-cells and cardiomyocytes, a phenomenon termed lipoapoptosis.^5^

The starting point for the present investigation was our observation that intramyocellular lipid deposition is a common feature of non-ischemic heart failure^6^ and that intramyocellular lipid droplets disappear from skeletal muscle after bariatric surgery.^7^ A key player in metabolic remodeling of liver, heart, and skeletal muscle is autophagy. More specifically, autophagy also regulates lipid metabolism, as first described in hepatocytes, where enhanced autophagy facilitates the breakdown of lipid droplets, reduces lipid accumulation, and is cytoprotective.^8^

**Figure 8:**
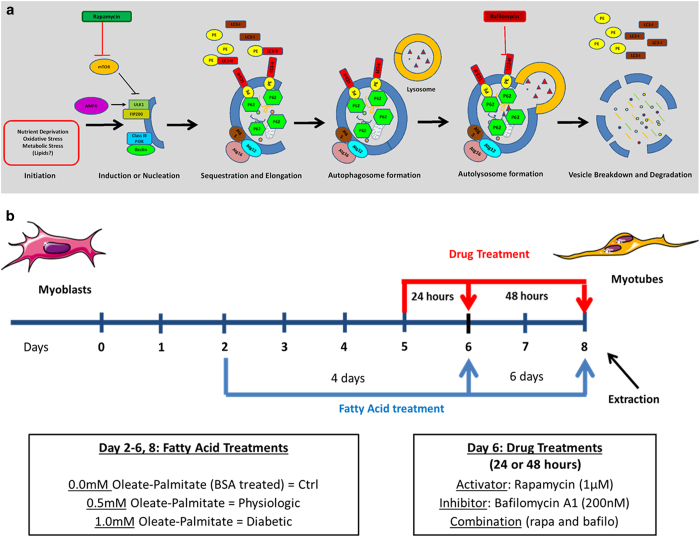
Modulation of autophagy and experimental design. (**a**) Autophagy is triggered by various stresses, including, but not limited to, nutrient deprivation, oxidative stress, and metabolic stress. To assess autophagic flux, we measured levels of two proteins: LC3, microtubule-associated protein light chain 3, and p62/SQSTM1 (Sequestosome1 complex), an adapter protein which acts as a substrate for the autophagy pathway. Two drugs, rapamycin and bafilomycin A1, were utilized to activate and inhibit the autophagy pathway, respectively. The autophagy pathway proceeds through six major steps: (1) Initiation, (2) Induction, (3) Elongation, (4) Autophagosome formation, (5) Autolysosome formation, and (6) Vesicle breakdown and degradation. (**b**) Summary of experimental design. Cells were treated with various fatty acid concentrations (0.0 mM oleate/palmitate=Control; 0.5 mM oleate/palmitate=Physiologic; 1.0 mM oleate/palmitate=Diabetic) and either rapamycin (1 uM), bafilomycinA1 (200 nM) drugs, or a combination of both.

A major consequence of pharmacological or nonpharmacological treatment of insulin resistance in obesity is the disappearance of triglycerides (TGs) from the heart and lipid droplets from skeletal muscle.^7,9,10^ The mechanisms underlying the disappearance of lipid droplets in muscle are at present not fully understood. The goal of this study was therefore to assess whether the loss of lipid droplets is the result of enhanced autophagic activity, as it has been described in the liver.^8^ In order to test this hypothesis, we used a skeletal muscle cell line (L6 myocytes) as a model system and modulated autophagic flux in the presence of lipid overload (Figure 8). Pharmacological activation of autophagy reversed lipid accumulation and decreased apoptosis. The opposite occurred with pharmacological inhibition of autophagy. Additionally, p62 associated with lipid droplets and transported the lipid droplets outside the cell. The results reveal a new pathway in the autophagic process of lipid droplet degradation and clearance from the cell.

## Results

### Pharmacological interventions promote the modulation of autophagy in L6 myocytes

**Figure 1:**
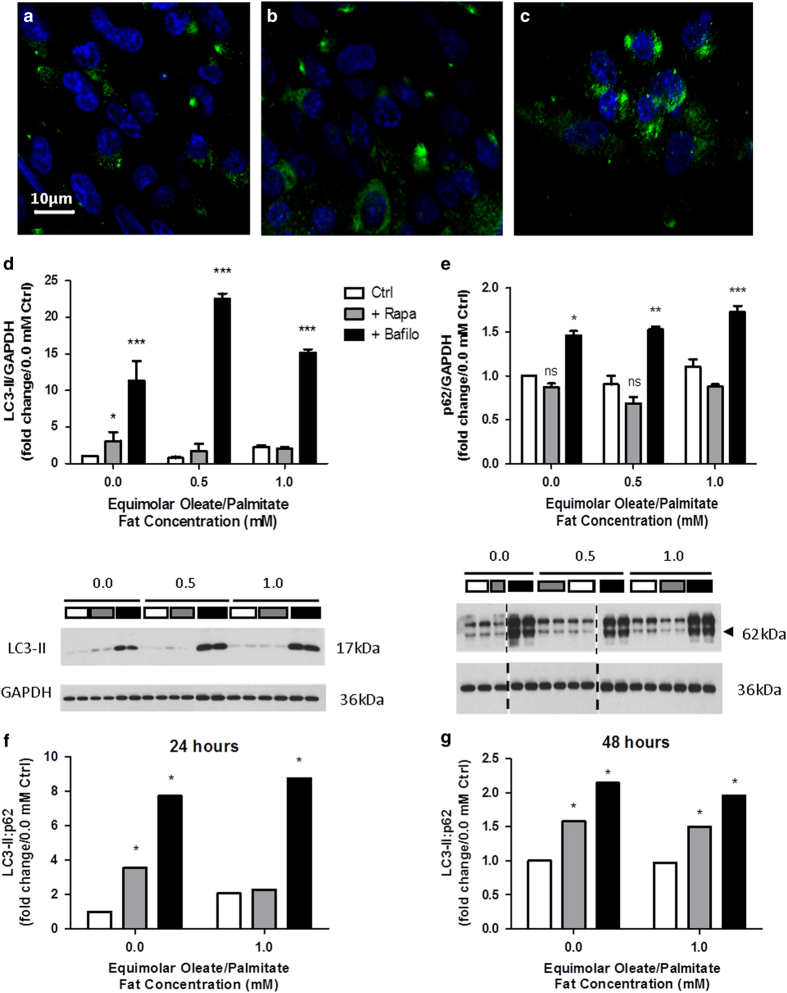
Activation of autophagy in L6 myocytes as measured by LC3-II and p62 protein levels; autophagic flux is higher in 48 h treated *versus* 24 h treated cells. (**a**–**c**) Immunofluorescence of cells treated with 1.0 mM oleate/palmitate fatty acids and without drugs (Ctrl) (**a**), with rapamycin (**b**), or with bafilomycin (**c**). All immunofluorescence images were taken at ×20 magnification. (**d**) LC3-II protein levels for these treatments. (**e**) p62 protein levels for these treatments. (**f** and **g**) LC3-II:p62 ratios of protein levels at 24 h (**f**) and 48 h (**g**). Ratios for the bafilomycin treatment in each graph represent inhibition of autophagy while ratios for control represent basal autophagy. Values in between are representative of autophagic flux. Data are expressed in fold change of protein levels *versus* BSA control at 0.0 mM oleate/palmitate (NS=not significant, *P<0.05, **P<0.01, ***P<0.001).

In order to assess whether pharmacological interventions promote the modulation of autophagy in L6 myocytes, we treated the cells with rapamycin or bafilomycin for 24 h and measured two protein markers of autophagy (LC3-II and p62) via immunofluorescence and immunoblotting (Figures 1a–g). Autophagic flux was assessed by measuring the LC3-II:p62 ratio, as previously reported by others.^11,12^ Immunofluorescence of cells treated with rapamycin revealed an increase in LC3-positive puncta confirming an increase in autophagy compared with 1.0 mM Ctrl cells (Figures 1a and b). Further increase of LC3 puncta in bafilomycin-treated cells indicated the impairment of autophagy and failure to degrade the autophagosome (Figure 1c). Immunoblotting confirmed activation of autophagy by rapamycin with increased LC3-II protein levels (Figure 1d) in conjunction with decreased p62 levels (Figure 1e). Taken together, an increased LC3-II:p62 ratio would suggest an increase in autophagic flux (Figures 1f and g). Treatment with bafilomycin yielded the highest LC3-II:p62 ratio, representing the inhibition of autophagic flux. Therefore, LC3:p62 ratio higher than that of Ctrl and lower than that of bafilomycin-treated cells would be indicative of true autophagic flux.

The inhibitory effect of bafilomycin was more pronounced at 24 h after treatment, while rapamycin induced a higher activation of autophagic flux after 48 h (Figures 1f and g). It is noteworthy that the presence of fatty acids was linked to the ability of rapamycin to increase autophagy. In the 1.0 mM, 24 h treatment group, rapamycin had no significant effect on autophagy when compared with the control group (without drugs) (Figures 1d–f). However, at 48 h, and in the presence of fatty acids, rapamycin significantly increased autophagic flux. Therefore, the optimal duration of treatment to observe a significant effect with both rapamycin and bafilomycin was 48 h. Accordingly, in all subsequent experiments, L6 myocytes were treated with drugs for 48 h as well.

### Rapamycin and bafilomycin have opposing effects on TG accumulation

**Figure 2:**
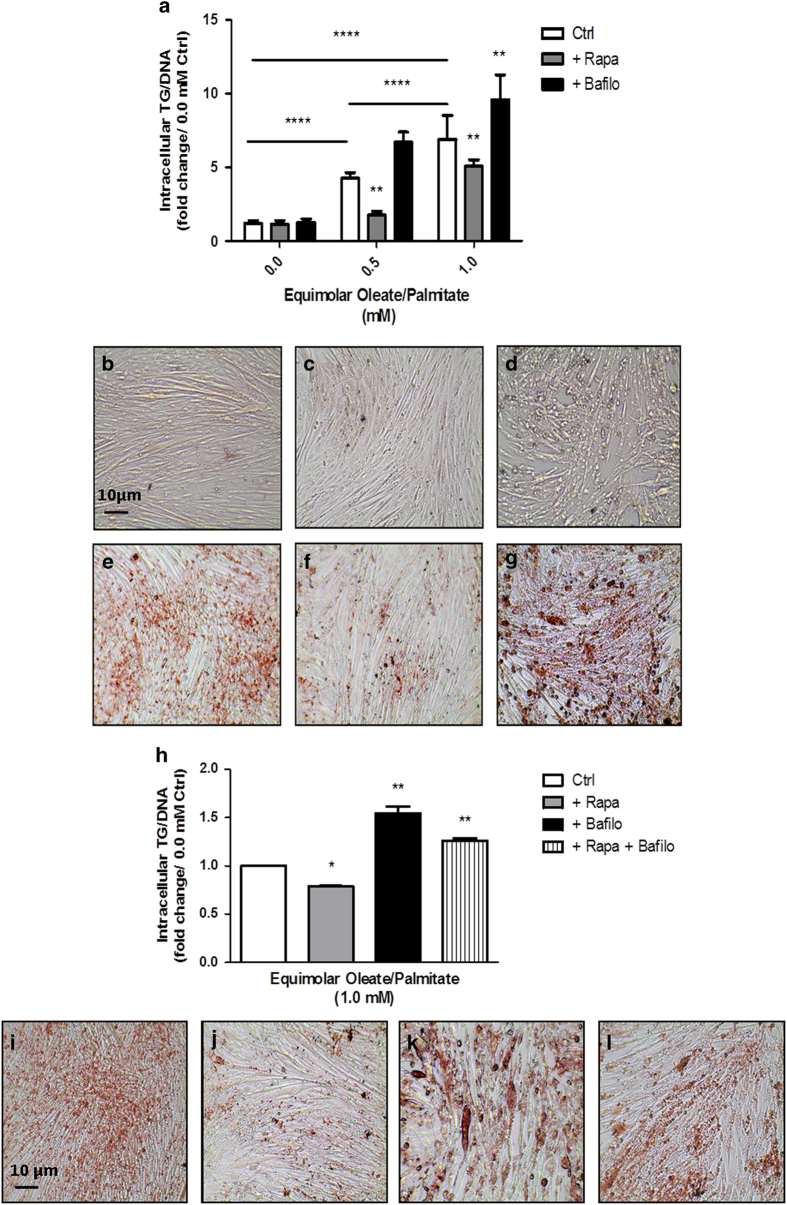
TG levels increase with bafilomycin and decrease with rapamycin treatment; combination treatment decreases TG levels as well. (**a**–**g**) Measurement of intracellular TG for cells treated with drugs for 24 h and (**h**–**l**) for 48 h. L6 myocytes were treated with changing levels of fat and measured by colorimetric assay (**a** and **h**) and assessed by Oil Red O staining (**b**–**g**) and (**i**–**l**). (**b**–**d**) All cells treated with BSA (0.0 mM) with no drugs (**b**), with rapamycin (**c**), or with bafilomycin (**d**). (**e**–**g**) and (**i**–**l**) All cells treated with 1.0 mM oleate/palmitate fatty acids with no drugs (**e** and **i**), with rapamycin (**f** and **j**), with bafilomycin (**g** and **k**), or with both rapamycin and bafilomycin (**l**). All images were taken at ×20 magnification (*P<0.05, **P<0.01, ****P<0.0001).

Next we investigated whether rapamycin and bafilomycin differentially affected TG accumulation in L6 myocytes. As expected, TG increased with increasing concentration of fatty acids in the culture medium (Figure 2a). Rapamycin decreased intracellular TG accumulation at both concentrations of fatty acids (0.5 and 1.0 mM) used. Conversely, bafilomycin dramatically increased intracellular TG levels when compared with the other treatment groups (Figure 2a). The efficiency of rapamycin in TG accumulation appeared to be dependent on the concentration of fatty acids used. Although rapamycin reduced TG accumulation by 59% in cells treated with 0.5 mM fatty acids, it only decreased TG accumulation by 26% in the 1.0 mM group. Oil Red O staining mirrored the TG assay data (Figures 2b–g), revealing a significant decrease in TG with rapamycin treatment (Figure 2f) and a dramatic increase in TG in the 1.0 mM and bafilomycin treatment (Figure 2g). Taken together, the results suggest that the activation of autophagy is involved in removal of TG from myocytes; the inhibition of this process impairs lipid removal and leads to a dramatic increase in TG. Moreover, there is an apparent decrease in cell number and change in morphology of cells treated with bafilomycin (Figures 2d and g). These findings suggest decreased cell viability, prompting us to investigate whether impaired autophagy promoted lipoapoptosis.

### Lipid-induced cell death (lipoapoptosis) is determined by intracellular TG content and levels of autophagic flux

**Figure 3:**
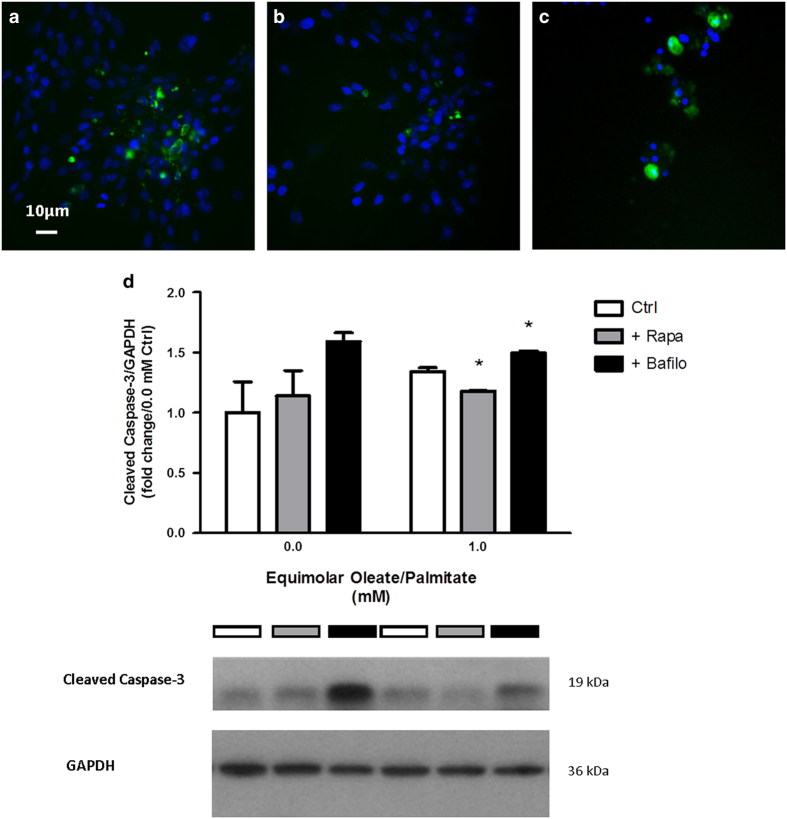
Rapamycin decreases whereas bafilomycin promotes cell death in the presence of fat. (**a**–**c**) Immunofluorescence of same cells treated with (**a**) no drugs, (**b**) rapamycin, and (**c**) bafilomycin. (**d**) Cleaved caspase-3 protein levels for cells treated with (1.0 mM) or without (0.0 mM) oleate/palmitate fatty acids with no drug, rapamycin, or bafilomycin. DAPI stains for nuclei while FITC stains for cleaved caspase-3 proteins. All immunofluorescence images were taken at ×20 magnification. Immunoblotting data are expressed in fold change of protein levels *versus* BSA control at 0.0 mM oleate/palmitate (*P<0.05).

In order to determine whether apoptosis is modulated by intracellular TG content and levels of autophagic flux, we measured protein levels of cleaved caspase-3 by immunofluorescence and immunoblotting (Figure 3). When cells were treated with rapamycin, cleaved caspase-3 levels were lower than that of control cells (Figures 3a and b). Conversely, cells treated with bafilomycin showed high levels of cleaved caspase-3, suggesting that apoptosis has been activated in these cells (Figure 3c). Decreased nuclear staining for the cells treated with bafilomycin indicated that a large number of cells were already dead at the time immunostaining was performed. Immunoblotting for cleaved caspase-3 protein levels confirmed the results (Figure 3d). This suggests that the activation of autophagy in lipid-laden myocytes is protective against programmed cell death, whereas inhibition of autophagy aggravates it.

### Treatment with a combination of rapamycin and bafilomycin suggests an alternative pathway for the breakdown and removal of fat from myocytes

**Figure 4:**
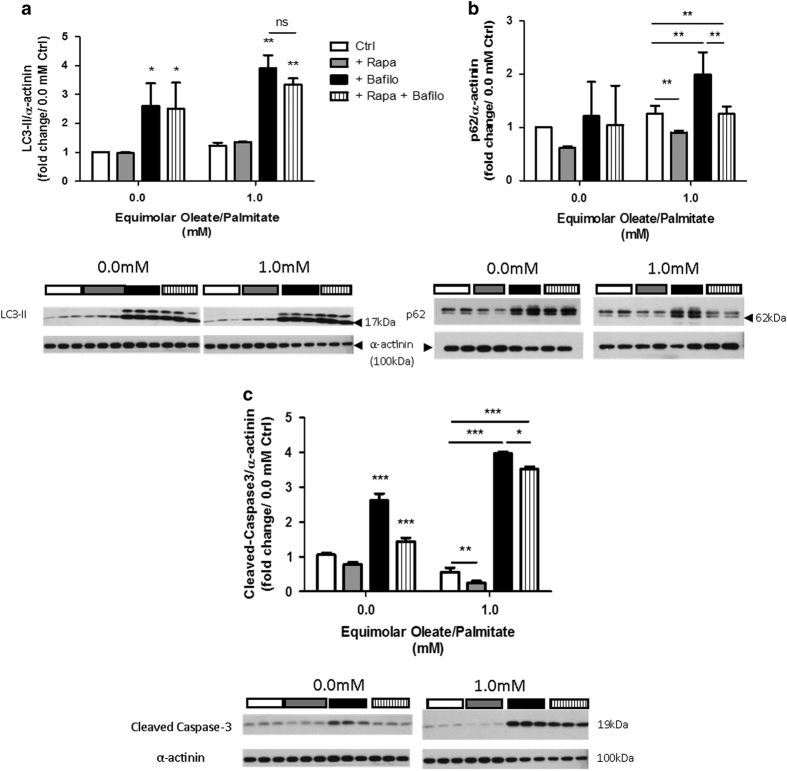
Combination of rapamycin and bafilomycin treatment suggest a new mechanism for clearing intramyocellular lipids. L6 myocytes were treated with rapamycin, bafilomycin, and a combination of both rapamycin and bafilomycin. (**a**) LC3-II, (**b**) p62, and (**c**) cleaved caspase-3 protein levels. Data are expressed in fold change of protein levels *versus* BSA control at 0.0 mM oleate/palmitate (*P<0.05; **P<0.01; ***P<0.001; NS, not significant).

**Figure 5:**
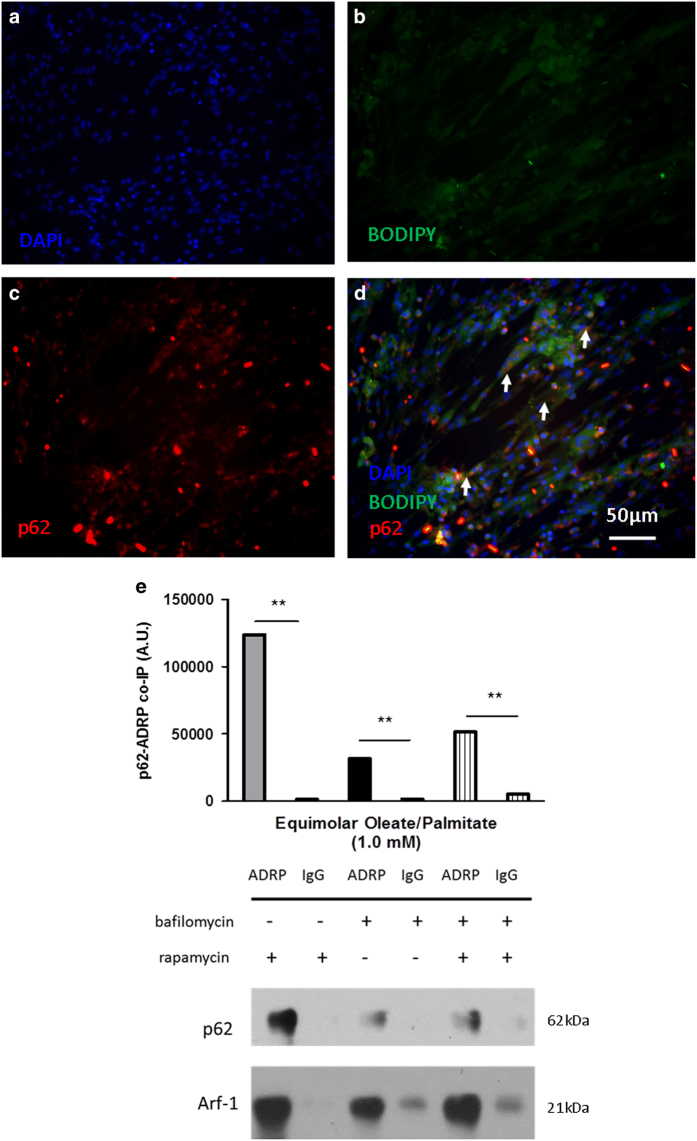
p62 associates with lipid droplets with the combination treatment of both rapamycin and bafilomycin. Immunofluorescence of cells stained with (**b**) BODIPY 493/503 (neutral lipid stain) and (**c**) TRITC (p62). (**a**) DAPI was used for nuclear staining. Cells were treated with both rapamycin and bafilomycin. White arrowheads in panel (**d**) indicate areas of colocalization between p62 and lipid droplets. All images were taken at ×40 magnification. (**e**) Immunoblotting for co-immunoprecipitation between p62 and ADRP in the presence of rapamycin, bafilomycin, or both. IgG groups represent negative controls; immunoblotting for Arf-1, known co-immunoprecipitate of ADRP, represent the positive controls. All cells were treated with 1.0 mM oleate/palmitate (**P<0.01).

**Figure 7:**
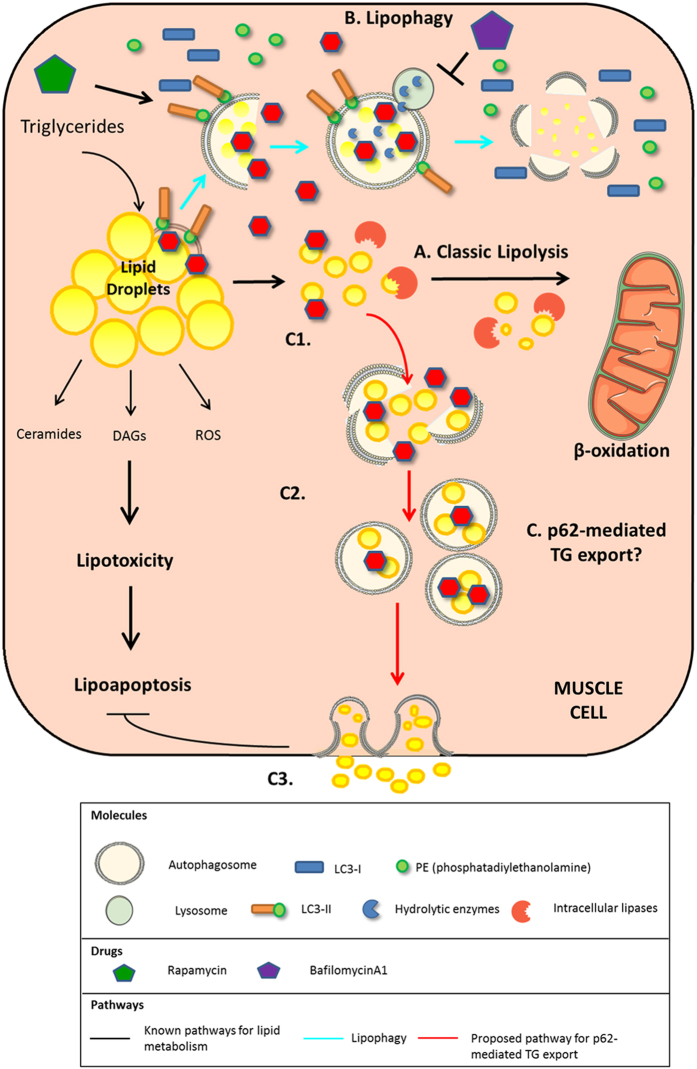
A proposed pathway for lipid removal from the myocyte. Muscle cells have been shown to breakdown lipid droplets through the classical pathway of lipolysis (A) or more recently by lipophagy (B). When cells are treated with high amounts of fat for a long period of time or lipophagy is impaired by a lysosomal defect, a new lysosomal-independent pathway for lipid removal is activated. P62 binds to lipid droplets (C1) and eventually leads to the export of TGs into the extracellular space (C3). The mechanism by which the TGs are being transported outside the cell is unknown and is currently under investigation (C2).

Another method for determining flux is by assessing the turnover of both LC3-II and p62 with simultaneous downstream inhibition (bafilomycin) and upstream activation (rapamycin) of autophagy (Figures 7 and 8a). The inhibition of autophagy at the autophagosomal–lysosomal fusion step, while ramping up the process at the induction step, allows one to assess whether the accumulation of LC3-II owing to rapamycin is a result of increased autophagy rather than impaired breakdown of the autophagosome.^13^ Under conditions of flux and the rapamycin and bafilomycin treatment, one would expect LC3-II and p62 protein levels to be similar to or increase beyond that of cells treated with bafilomycin only. Although this was the case for the 0.0 mM group, this was not the case in the 1.0 mM group. Although LC3-II levels were comparable for both treatments (Figure 4a), p62 levels were different (Figure 4b). Treatment with both drugs would be expected to increase p62 levels as well (considering that p62 can no longer be degraded by the lysosomal machinery). However, levels of p62 in this group were similar to the 1.0 mM control group (Figure 4b). Decreased cleaved caspase-3 levels suggested a decrease in apoptosis for the rapamycin- and bafilomycin-treated cells compared with the bafilomycin-only-treated cells (Figure 4c). This result was not totally unexpected, because in the same treatment group TG levels were also decreased, as evidenced by the TG assay measurements and Oil Red O staining (Figures 2h–l). The results suggest that activation of autophagy via rapamycin is only protective against cell death, which occurs in the presence of fat. Immunofluorescence probing for both p62 and neutral lipids (BODIPY) showed colocalization of these two molecules in the presence of 1.0 mM oleate/palmitate fatty acids and the combination treatment of rapamycin and bafilomycin (Figures 5a–d). Moreover, this occurred in spite of sequestration of p62 into the autophagosome (Figure 8). The unexpected decrease in intramyocellular lipid accumulation, the decrease in p62 levels, and the colocalization of p62 to lipid droplets in rapamycin- and bafilomycin-treated cells all suggest a novel pathway for the breakdown of lipids.

### p62 interacts with lipid droplets at a specific membrane protein

To confirm that p62 indeed colocalized to lipid droplets, we performed co-immunoprecipitation experiments probing for p62-ADRP aggregates (Figure 5e). ADRP, also known as perilipin 2 (PLIN2), is the predominant lipid droplet membrane protein in skeletal muscle. Cells treated with rapamycin revealed a high amount of interaction between p62 and ADRP in the presence of fat, suggesting that activation of autophagy may also stimulate p62 colocalization with lipid droplets. Interestingly, there was significant co-immunoprecipitation as well in the rapamycin and bafilomycin group, albeit the interaction was only half of that in the rapamycin only group. The bafilomycin group also indicated some p62-ADRP co-immunoprecipitation. This group, however, yielded the lowest interaction compared with the other two groups. The results from the co-immunoprecipitation experiments suggest that p62 specifically interacts with lipid droplets at the membrane in the presence of fat and activation of autophagy.

### The inhibition of cytosolic lipases does not affect the impact of autophagy modulation on intracellular TG removal

**Figure 6:**
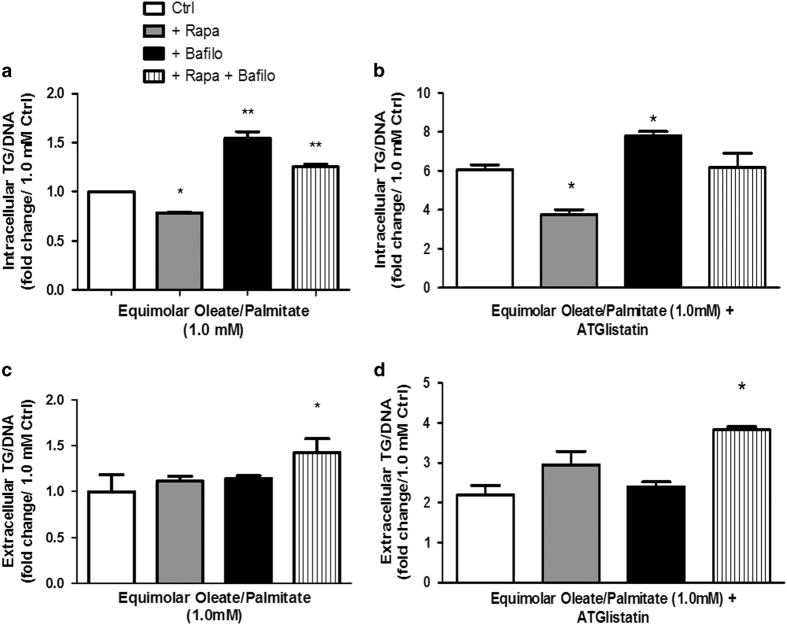
Extracellular TG reveals potential mechanism for export of TG; ATGListatin does not alter effects of autophagy drugs. Amount of TGs (**a** and **b**) in the cells and (**c** and **d**) in the extracellular space after treatment with the various drugs. Intracellular and extracellular TG were measured from cells treated with ATGListatin in addition to autophagy drugs (**b** and **d**). Data are expressed in fold change of protein levels *versus* corresponding 1.0 mM oleate/palmitate Ctrl value intracellular (**a**) or extracellular (**c**) (*P<0.05, **P<0.01).

To investigate whether the neutral lipids were broken down by cytosolic lipases, we added ATGListatin, a pharmacological inhibitor of adipose triglyceride lipase (ATGL) (Figures 6b and d). If the pathway induced by the combination treatment of rapamycin and bafilomycin proceeded through lipolysis, inhibition of lipolysis would affect lipid accumulation for these cells. As expected, intracellular TG levels were increased with ATGListatin treatment compared with levels for cells treated with autophagy drugs, but without ATGListatin (Figures 6a and b). The relative effects on TG accumulation between the various treatment groups were maintained, as evidenced by lower TG levels with rapamycin, higher TG levels with bafilomycin, and an intermediate amount of TG for the combination treatment (Figure 6b). The diminished role of lipolysis in lipid clearance in the cells subjected to the combination treatment and the inability of ATGListatin to alter the effects of pharmacological activation and inhibition of autophagy on intracellular TG levels both suggest a new pathway for the clearance of lipids that is independent of cytosolic lipase activity.

### Treating L6 myocytes with both rapamycin and bafilomycin promotes the export of fat

It is already known that heart muscle secretes TGs^14^ in order to secrete lipoproteins in a reverse TG transport manner, possibly to remove toxic lipids from the heart.^15^ As seen in the Oil Red O staining and TG assay earlier (Figures 2h and l), the combination of rapamycin and bafilomycin treatments decreases intracellular TG accumulation when compared with bafilomycin treatment alone (Figures 2h and k). However, as the TGs in this treatment group were not being broken down by cytosolic lipases, we wondered whether the TGs were somehow being secreted outside the cell. Using the same TG assay as before to measure intracellular TG, we measured the culture medium for extracellular TG (Figure 6c). Interestingly, the TG content in the culture medium of the rapamycin- and bafilomycin-treated cells was indeed considerably higher than in the medium of control or bafilomycin-treated cells (Figure 6c). Because the cells with the combination drug treatment were incubated with fatty acids and no TG, the results show that fatty acids were converted into TG and exported into the extracellular space. However, bafilomycin treatment did not result in lower levels of TG in the culture medium. This was an unexpected result given the difference in intracellular lipid accumulation between rapamycin- and bafilomycin-treated cells because one would expect for the bafilomycin-treated cells to have lower TG levels in the medium. Therefore, to determine whether this may be mediated by increased lipolysis, TG levels were also measured in the same cells with an additional treatment of ATGListatin (Figure 6d). In these cells, the combination treatment still yielded higher levels of medium TG despite the presence of an ATGL inhibitor. This suggests that the TG in this treatment group are not being processed by cytosolic lipases, but are instead exported into the extracellular space. The results imply that the pathway through which TG are exported from the cell is unique to the pharmacological combination treatment of rapamycin and bafilomycin and independent from lipolysis. We therefore propose that the simultaneous activation and inhibition of autophagy in the presence of high fat results in TG export from myocytes.

## Discussion

### Role of lipophagy in muscle

We have shown that autophagy is essential for degradation of lipid droplets in skeletal muscle myotubes. Inhibiting this process pharmacologically leads to dramatic TG accumulation, whereas activating this process pharmacologically with rapamycin leads to the increased removal of TGs from the cell. Although alternative pathways for the breakdown of lipid droplets exist, autophagy appears to be required to remove intramyocellular TGs from the myocytes. Furthermore, the process is protective against cell death associated with lipid overload (lipoapoptosis) because promoting autophagy reduced lipid-induced cell death, whereas inhibiting autophagy increased cell death in the presence of high fat.

Singh *et al*.^8^ first described the role of autophagy in lipid metabolism in cultured hepatocytes and in the mouse liver. Inhibition of autophagy both pharmacologically and genetically in these systems led to accumulation of TGs and subsequently lipid droplets. Furthermore, inhibited autophagy led to decreased *β*-oxidation, suggesting the involvement of autophagy in lipolysis and TG hydrolysis. Autophagy itself can be inhibited by lipids as well. For example, mice fed a high-fat diet for 16 weeks show decreased association of LC3 with lipid droplets (decreased autolipophagosomes) and therefore exhibit dampening of the autophagy pathway in the presence of an exogenous lipid load.^8^

We have also confirmed in myocytes the findings in hepatocytes reported by Singh *et al*.^8^ Impairment of autophagy leads to accumulation of intramyocellular TGs, whereas promoting autophagy facilitates removal of TGs from the cell. The excess accumulation of lipids owing to impaired autophagy leads to lipid-induced cell death. When autophagy is activated, lipid-induced cell death decreases. Another finding that matches those of Singh *et al*.^8^ is that autophagy was slightly suppressed in myocytes treated with fat for 24 h (Figure 1f). The phenomenon described in the liver may be one that occurs in other tissues as well and would be important to consider when using autophagy as a therapeutic target for treating conditions of dysregulated lipid metabolism.

Our studies have yielded some unexpected results. When lipid-overloaded cells were treated with a combination of both an activator and an inhibitor of autophagy, rapamycin and bafilomycin, respectively, there was a surprising and dramatic decrease in lipids. We therefore began to investigate autophagic molecules that could drive this process upstream of the autophagolysosome formation and identified p62 (Figure 7). The present colocalization experiments provide evidence for a previously unobserved p62–lipid droplet interaction. This process may be an adaptive mechanism for the cells, allowing them to continue undergoing lipid clearance despite lysosomal dysfunction. The presence of ongoing p62-mediated lipid removal under conditions of a lysosomal defect may prove to be an essential signaling pathway for the discovery of new drugs associated with lysosomal storage disorders.

### Lipid metabolism and lipotoxicity

Biochemically, lipid metabolism in skeletal muscle is a highly regulated process. It has already been shown that proteins on the sarcolemma (e.g., FATP, FAT/CD36) are responsible for the uptake of fatty acids into the skeletal muscle cell.^16^ Once inside, long-chain fatty acids are bound to fatty acid-binding protein and are converted to acyl-CoA by acyl-CoA synthetase (ACS). ACS can also mediate the synthesis of acyl-CoAs from intramuscular lipolysis by ATGL, hormone-sensitive lipase, and monoacylglycerol lipase; the acyl-CoAs are then transported into the mitochondria via the carnitine palmitoyltransferase shuttle for *β*-oxidation and the production of ATP.^17^ However, when uptake of FFAs exceeds anabolic processes of the cell, lipids are esterified and stored as TGs in lipid droplets.^18^ Skeletal muscle has a limited storage capacity for lipids; when this limit is exceeded, lipids enter alternative non-oxidative pathways that result in the production of toxic reactive lipid species and ultimately lipid-induced dysfunction (lipotoxicity) or even cell death (lipoapoptosis).^3^

Lipotoxicity and lipoapoptosis in nonadipose tissues leads to derangement of cellular structure and function.^6^ For example, lipotoxicity impairs myocardial contractility in obesity.^19^ This functional loss is specifically associated with the loss of cardiomyocytes by apoptosis.^3^ In skeletal muscle, this excess intramyocellular lipid accumulation is associated with insulin resistance and oxidative stress.^18^ In a lipotoxic state, the inability to oxidize fatty acids in the skeletal muscle may be a consequence of reduced or impaired mitochondrial function.^16^ When the muscle is overloaded with fat, the autophagy pathway is suppressed.^8^ Turning this pathway back ‘on’ in our *in vitro* model leads to the decreased accumulation and breakdown of intramuscular fat. This is ultimately beneficial for the cell and leads to increased survival of cells despite the presence of high fat. The converse is true as well. Here we have shown that further inhibition of autophagy leads to further accumulation of lipids and evidence of lipotoxicity.

### A new pathway for lipid clearance

Our work also suggests that a pathway exists that exports lipids outside the myocyte. Two pathways that are responsible for trafficking substrates outside the cell are the caveolar pathway and exocytosis. Caveolae have been shown to interact with cholesterol, bind to fatty acids, and associate with lipid droplets.^20^ Various studies have shown that a caveolin truncation mutant decreased surface levels of free cholesterol and has increased neutral lipid storage in lipid droplets. However, the mechanisms by which this occurs is still unclear.

With regards to exocytosis, observations by others have shown that the shuttling of lipids outside the cell via exocytosis is utilized for cell-to-cell communication^21^ and triggered by cell therapy.^22^ Autophagy and exocytosis are also closely associated in different, but sometimes complementary lysosomal processes. In the brain, autophagic failure prompts the cell to switch to exocytosis for the clearance of alpha-synuclein aggregates. This is ultimately detrimental because it deposits alpha-synuclein onto neighboring cells and causes neuronal apoptosis.^23^ In Pompe’s disease, however, exocytosis works with autophagy to promote transcription factor EB (TFEB)-mediated cellular clearance.^24^ TFEB is known to regulate the lysosomal machinery in a cell. Feeney *et al*.^24^ have shown that overexpression of TFEB in muscle cells of Pompe’s disease causes exocytosis of autolysosomes. The exocytosis of lipids as well as the previously established relationship between autophagy and exocytosis together suggests the possibility of a lipophagy-mediated exocytosis, as our experiments seem to point to. The lipid removal mechanism we have discovered is involved in the clearance of potentially harmful aggregates, much like in the alpha-synuclein studies from Lee *et al*.^23^ The process also utilizes the autophagy machinery for clearance of cellular substrates, much like in the muscle cell studies from Feeney *et al*.^24^ This ultimately brings to light a p62-mediated exocytosis pathway for clearance of lipids that involves a lysosomal-independent, autophagy process. The new question, however, is: What effects does this lipid exportation process have on neighboring cells? Getting rid of TGs from the cell may be protective for the cell itself, but perhaps the TG will be deposited in other cells, where they can be harmful, as implicated by the studies of alpha-synuclein deposition in neighboring neurons.^23^ Perhaps, this exocytosis mechanism can generate exosomes that communicate with other cells the presence of high TGs in neighboring cells, much like in the studies from Record *et al*.^21^ Although the process is still largely unknown, the potential for reversing lipotoxic diseases by upregulating p62-mediated TG export provides a new discovery platform for the development of drugs used to treat lipotoxicity in the muscle.

## Conclusion

Our experiments propose a new pathway that may, in part, explain how lipids are removed from the muscle cell after bariatric surgery. Classical autophagy is initially effective at removing potentially toxic lipids from the myocyte. However, when lysosomes become defective in conjunction with chronic lipid overload, the autophagy pathway is impaired and the cell must rely on an alternative pathway to clear lipids. The discovery of this lipid-clearing pathway in the myocyte changes our understanding of the reversal of intramyocellular lipid accumulation after weight loss surgery. Among the mechanisms for removing fat from the cell, the caveolae pathway and lipid exocytosis should be considered when exploring treatment for obesity and lipotoxicity. Future studies are needed to understand the metabolic effects of these pathways both on the cells themselves and on their environment to determine whether modulation of this pathway is safe and effective.

## Materials and Methods

### Reagents and antibodies

Rapamycin and Bafilomycin A1 were purchased from LC Laboratories (Woburn, MA, USA). Rapamycin activates autophagy upstream of the lysosomal fusion step by inhibiting mTORC1 (mechanistic Target of Rapamycin Complex 1), which is an inhibitor of autophagy at ULK1.^25^ Bafilomycin A1 inhibits autophagy further downstream by preventing the fusion of the lysosome to the autophagosome at the vacuolar H^+^ ATPase receptor on the lysosome^26^ (Figures 7 and 8). ATGListatin was purchased from Cayman Chemical (Ann Arbor, MI, USA). ATGListatin was used to inhibit lipase activity in the ATGL enzyme. The colorimetric assay used for determination of TG in the samples was purchased from Wako Diagnostics (Waco, VA, USA); the kit used here is the Wako L-Type TG H Assay Kit (Reagent 1 no. 461–09092, Reagent 2 no. 461–08992, and Lipids Calibrator 464–01601) (Wako Diagnostics). As antibodies for the assessment of autophagy, we used p62/Anti-SQSTM1 (1 : 5000) from AbNova (UTEMB020, Walnut, CA, USA) and LC3 (1 : 1000) from Novus Biologicals (NB100–2220, Littleton, CO, USA). Alpha-actinin (1 : 500) (3134) and caspase-3 (1 : 1000) (9662) were purchased from Cell Signaling Technology (Danvers, MA, USA). GAPDH (glyceraldehyde 3-phosphate dehydrogenase) (1 : 30 000) was purchased from Fitzgerald (10R-g109a, Acton, MA, USA). ADRP (H-80) antibody (sc-12888) used for co-immunoprecipitation (2 *μ*g: 300 *μ*g of protein to 200 *μ*l of cell lysate) was purchased from Santa Cruz Biotechnology (Dallas, TX, USA). Arf-1 (1 : 1000) antibody was purchased from Novus Biologicals. Normal rabbit IgG was purchased from Cell Signaling Technology and matched the concentration of the test antibody for the co-immunoprecipitation experiments. Anti-rabbit (sc-2004) and anti-mouse (sc-2005) secondary antibodies (both diluted to 1 : 2000) were also purchased from Santa Cruz Biotechnology. All antibodies were prepared in a solution of 5% (w:v) nonfat dry milk in 1× TBS with 0.4% Tween 20. The PBS (phosphate-buffered saline) used throughout the experiments do not contain CaCl or MgCl.

### Cell culture and experimental design

L6 rat myogenic cells were cultured in growth medium composed of DMEM containing l-glutamine (584 mg/l), sodium pyruvate (110 mg/l), and d-glucose (1.0 g/l) and supplemented with 10% fetal bovine serum, penicillin (100 units/ml), and streptomycin (100 *μ*g/ml). Once they reached confluence, the cells were switched to a differentiation medium composed of DMEM supplemented with 2% horse serum, penicillin (100 units/ml), and streptomycin (100 *μ*g/ml). Sodium oleate and sodium palmitate were conjugated to BSA, Cohn Fraction V, and charcoal-treated (Probumin, Darmstadt, Germany) as previously described.^27^ Briefly, sodium palmitate is dissolved in NaOH (0.05 M) solution prepared in PBS while sodium oleate is dissolved in PBS alone to produce 50 mM stock solutions of fatty acids. Both stock solutions were dissolved at 60 °C for about 10–15 min with periodic vortexing. A separate BSA solution (18.3% w–v) was prepared by dissolving BSA in cell culture medium. Each 50 mM fatty acid solution was added dropwise to the BSA at an equimolar 2 : 1 ratio to obtain a 5 mM oleate/palmitate stock solution. The stock was sterile filtered using a 0.22 *μ*m Steriflip vacuum filtration system (EMD Millipore Corporation, Darmstadt, Germany). Prior to treatment, the stock was diluted in warm, differentiation medium to produce fatty acid mixtures at 0.5 or 1.0 mM concentrations. The cells were differentiated for 2 days before treatment with a 0.5 or 1.0 mM equimolar mixture of oleate and palmitate or BSA alone (control) for a period of 4–6 days (Figure 8). Medium was changed every 2 days. During the last 24–48 h of incubation, cells were treated with rapamycin (1 uM), bafilomycin (200 nM), or a combination of both to assess the effects of autophagy activation and inhibition, respectively, on skeletal muscle lipid metabolism. The concentrations for the drugs were adapted from a similar study using a C2C12 skeletal muscle cell line.^11^ For inhibition of ATGL, ATGListatin (10 *μ*M) was administered 24 h before autophagy drugs. All drugs were dissolved in DMSO. Cells fixed for microscopy were rinsed with ice-cold PBS and then immersed in paraformaldehyde (4%) (w:v) for 10–12 min; after incubating the cells in paraformaldehyde, cells were then washed again with ice-cold PBS before staining the cells.

### Imaging

#### Immunofluorescence

After 2 days of differentiation, cells were trypsinized and re-seeded at 30% density on chambered slides treated with Permanox (Thermoscientific, Waltham, MA, USA). Before fixation, medium was removed and the slides were washed with cold PBS twice. Cells were fixed at room temperature in 4% paraformaldehyde (w:v) prepared in cold PBS for 12–15 min. Afterwards, slides were washed twice with cold PBS again and were kept at 4 °C overnight in preparation for staining. Blocking was achieved by incubating wells with goat serum (10%) (v:v) in PBS for 20 min. Primary antibodies (LC3, 1 : 1000; p62, 1 : 2000; cleaved caspase-3, 1 : 500) were diluted in goat serum (3%) (v:v) in PBS; BODIPY (493/503) was prepared at 1 mg/ml (w:v) in PBS. Slides were then washed in cold PBS once and then incubated with the appropriate antibodies overnight at 4 °C and/or BODIPY at room temperature for 4 h. Next cells were incubated in secondary antibody (Alexa Fluor Goat anti-rabbit or Alexa Fluor Goat anti-mouse at 1 : 500) 1 h at 37 °C, before applying the mounting medium (Prolong Gold Antifade Mounting media with DAPI, ProLong, Grand Island, NY, USA) and coverslip. Samples were allowed to cure overnight in the dark at 4 °C and then analyzed by either epifluorescence microscope (Nikon Eclipse Ti-E, Nikon, Melville, NY, USA) or confocal microscope (Nikon a1r, Nikon).

#### Oil Red O staining

Oil Red O Stock solution was prepared by mixing Oil Red O powder (Sigma-Aldrich, St Louis, MO, USA) (500 mg : 100 ml, w–v) in isopropanol. A working solution of 60% (v:v) ORO was prepared fresh by diluting in distilled water. Cells were rinsed with ice-cold PBS once then treated with paraformaldehyde (4%) (w:v) in PBS for 10 min at room temperature. Thereafter, the paraformaldehyde was then removed, cells were washed with cold PBS twice, and ORO solution (60%) was added to each of the cells. Cells were incubated in the ORO working solution for 15 min, rinsed in ice-cold PBS twice, and dried before visualization under light microscopy (Motic AE2000, Motic, Richmond, BC, Canada).

### TG assay and DNA extraction

#### Enzymatic TG assay

Extraction and quantification of TGs from L6 myocytes was adapted from the method described by Bligh and Dyer.^28^ Cells were pelleted by centrifugation for 5 min at 10 000 r.p.m. The supernatant was aspirated from samples, and cell pellets were sonicated in Methanol/Chloroform (2 : 1, v–v). After sonication, samples were centrifuged for 3 min at 8000 r.p.m., and the supernatant for each sample was kept. Chloroform and KH_2_PO_4_ were then serially added to induce separation of the two phases (lower chloroform phase is total lipid fraction). After a final centrifugation step (3 min at 8000 r.p.m.), the supernatant is recovered, roto-evaporated in a centri-vap, and re-suspended in LPL buffer. The LPL buffer was prepared according to the recipe as described by Rodriguez-Sureda and Peinado-Onsurbe.^29^ Intracellular TG were then quantified with the L-Type TG H Assay Kit.

#### DNA extraction

The remaining upper phase from the TG extraction was kept for isolation and quantification of ssDNA for normalization of each of the samples. NaOH, 0.2 M, was added to the upper phase of each sample and the mixture was vortexed and inverted for 5 min. The samples were then heated at 60 °C for 10 min to ensure the complete lysis of the cell membrane. Measurements were made with a nanodrop spectrophotometer (BioTek Synergy, Winooski, VT, USA).

### Protein extraction and western blotting

Protein homogenates were prepared in the presence of inhibitors of phosphatases (Sigma-Aldrich) and proteases (Roche Applied Science, Penzberg, Germany). Protein concentration of the samples was measured using the Pierce BCA Protein Assay Kit (Thermo Scientific, 23227, Rockford, IL, USA). Proteins were detected by immunoblotting using horseradish peroxidase-conjugated secondary antibodies and chemiluminescence (Santa Cruz Biotechnology). Protein samples were ran on SDS-PAGE (12%) gels. Gels were blotted onto PVDF membranes by transfer at a constant 35 volts overnight at 4 °C. The membranes were then blocked by nonfat dry milk solution (5%) in 1× TBS (Tris Buffered Saline) with Tween 20 (0.4%) and incubated in desired primary antibody overnight. Membranes were then washed in 1× TBS with Tween 20 (0.4%) three times, 5 min each before incubating with the secondary antibody for 1 h at room temperature. All densitometric analyses for western blottings were performed using ImageJ 1.46r (NIH, Bethesda, MD, USA).

### Autophagic flux determination

Autophagic flux was estimated from the turnover of LC3-II and p62 as previously shown.^11,30^ Briefly, protein levels of LC3-II and p62 were measured by immunoblotting. The bafilomycin treatment represents a control for inhibition of autophagic flux, as indicated by the high LC3-II:p62 ratio, whereas the LC3-II:p62 ratio for the samples without drug treatment represent basal autophagy. In cells treated with bafilomycin, autophagy is impaired leading to the accumulation of autophagic vacuoles containing LC3-II owing to failure of autophagosome–lysosomal fusion. This would cause a high LC3-II:p62 value. Cells without any drug treatment have low levels or basal autophagy, where p62 levels are high, as they are not being degraded by autophagy, and LC3-II levels are low, as autophagy is not being activated. This would create a low LC3-II:p62 value. These two treatment groups represent the upper and lower bounds, respectively, for assessing autophagic flux. Therefore, any LC3-II:p62 value between the values for control (no drug treatment) and the bafilomycin-treated cells represent the activation and completion of autophagy (autophagic flux).

### Co-immunoprecipitation

Cells were grown in six-well plates and sonicated in non-denaturing lysis buffer to prevent breakdown of protein–protein interactions. For 10 ml of protein extraction buffer, 2.5 ml Tris HCl (0.2 M, pH 7.5), 0.1 ml NP-40, 670 *μ*l NaCl (1.5 M), and 6.73 ml of ddH2O were mixed. Cells were sonicated in 500 *μ*l of cold lysis buffer for 15 s each. Next the cells were centrifuged for 20 min at 12 000 r.p.m. at 4 °C. The supernatant (protein) was aspirated, collected, and measured with the BCA assay, as mentioned earlier. Samples were incubated with desired primary antibody overnight at 4 °C. A total of 40 *μ*l of protein A/G PLUS agarose beads (50% bead slurry) (Santa Cruz Biotechnology) was added to each sample and was rocked gently for 80–90 min at 4 °C. Immunoprecipitates were collected by centrifuging the samples at 2500 r.p.m. for 5 min at 4 °C. Pellet was saved and washed with 500 *μ*l wash buffer, each time repeating the centrifugation step. Pellet was resuspended in 40 *μ*l loading buffer (2×) and boiled at 100 °C for 8 min to denature the proteins and separate it from the beads. Samples were vortexed and then centrifuged for 30 s and the supernatant was collected (proteins of interest). Beads in the pellet were discarded and the supernatant was used to prepare western blotting samples that were run on an SDS-PAGE and probed with other protein of interest.

### Statistical analysis

Values are given as the mean±S.E.M. The number of independent experiments and replicates is given in the figure legends. Statistical significance between values was determined by Student’s *t*-test or ANOVA to test the significance of variances, using Graphpad Prism 5 (La Jolla, CA, USA). A *P*-value <0.05 was considered to be significant.

## References

[bib1] Friedman J. Fat in all the wrong places. Nature 2002; 415: 268–269. 11796987 10.1038/415268a

[bib2] Virchow R. Cellular Pathology as Based Upon Physiological and Pathological Histology. Dover Publications: New York, USA, 1971; xxvii: 554 p.

[bib3] Unger RH. Lipotoxic diseases. Annu Rev Med 2002; 53: 319–336. 11818477 10.1146/annurev.med.53.082901.104057

[bib4] Slawik M, Vidal-Puig AJ. Lipotoxicity, overnutrition and energy metabolism in aging. Ageing Res Rev 2006; 5: 144–164. 16630750 10.1016/j.arr.2006.03.004

[bib5] Unger RH, Orci L. Lipoapoptosis: its mechanism and its diseases. Biochim Biophys Acta 2002; 1585: 202–212. 12531555 10.1016/s1388-1981(02)00342-6

[bib6] Sharma S, Adrogue JV, Golfman L, Uray I, Lemm J, Youker K et al. Intramyocardial lipid accumulation in the failing human heart resembles the lipotoxic rat heart. FASEB J 2004; 18: 1692–1700. 15522914 10.1096/fj.04-2263com

[bib7] Leichman JG, Wilson EB, Scarborough T, Aguilar D, Miller 3rd CC, Yu S et al. Dramatic reversal of derangements in muscle metabolism and left ventricular function after bariatric surgery. Am J Med 2008; 121: 966–973. 18954843 10.1016/j.amjmed.2008.06.033 PMC2604808

[bib8] Singh R, Kaushik S, Wang Y, Xiang Y, Novak I, Komatsu M et al. Autophagy regulates lipid metabolism. Nature 2009; 458: 1131–1135. 19339967 10.1038/nature07976 PMC2676208

[bib9] Zhou YT, Grayburn P, Karim A, Shimabukuro M, Higa M, Baetens D et al. Lipotoxic heart disease in obese rats: implications for human obesity. Proc Natl Acad Sci USA 2000; 97: 1784–1789. 10677535 10.1073/pnas.97.4.1784 PMC26513

[bib10] Unger RH, Orci L. Diseases of liporegulation: new perspective on obesity and related disorders. FASEB J 2001; 15: 312–321. 11156947 10.1096/fj.00-0590

[bib11] Ju JS, Varadhachary AS, Miller SE, Weihl CC. Quantitation of ‘autophagic flux’ in mature skeletal muscle. Autophagy 2010; 6: 929–935. 20657169 10.4161/auto.6.7.12785 PMC3039739

[bib12] Klionsky DJ, Abdelmohsen K, Abe A, Abedin MJ, Abeliovich H, Acevedo Arozena A et al. Guidelines for the use and interpretation of assays for monitoring autophagy, 3rd edn. Autophagy 2016; 12: 1–222. 26799652 10.1080/15548627.2015.1100356 PMC4835977

[bib13] Oh S, Xiaofei E, Ni D, Pirooz SD, Lee JY, Lee D et al. Downregulation of autophagy by Bcl-2 promotes MCF7 breast cancer cell growth independent of its inhibition of apoptosis. Cell Death Differ 2011; 18: 452–464. 20885445 10.1038/cdd.2010.116 PMC3017654

[bib14] Goodwin GW, Taylor CS, Taegtmeyer H. Regulation of energy metabolism of the heart during acute increase in heart work. J Biol Chem 1998; 273: 29530–29539. 9792661 10.1074/jbc.273.45.29530

[bib15] Bjorkegren J, Veniant M, Kim SK, Withycombe SK, Wood PA, Hellerstein MK et al. Lipoprotein secretion and triglyceride stores in the heart. J Biol Chem 2001; 276: 38511–38517. 11481337 10.1074/jbc.M106839200

[bib16] Holloway GP, Bonen A, Spriet LL. Regulation of skeletal muscle mitochondrial fatty acid metabolism in lean and obese individuals. Am J Clin Nutr 2009; 89: 455S–462S. 19056573 10.3945/ajcn.2008.26717B

[bib17] Zechner R, Kienesberger PC, Haemmerle G, Zimmermann R, Lass A. Adipose triglyceride lipase and the lipolytic catabolism of cellular fat stores. J Lipid Res 2009; 50: 3–21. 18952573 10.1194/jlr.R800031-JLR200

[bib18] Schaffer JE. Lipotoxicity: when tissues overeat. Curr Opin Lipidol 2003; 14: 281–287. 12840659 10.1097/00041433-200306000-00008

[bib19] Harmancey R, Wilson CR, Taegtmeyer H. Adaptation and maladaptation of the heart in obesity. Hypertension 2008; 52: 181–187. 18574077 10.1161/HYPERTENSIONAHA.108.110031 PMC3660087

[bib20] Parton RG, Simons K. The multiple faces of caveolae. Nat Rev Mol Cell Biol 2007; 8: 185–194. 17318224 10.1038/nrm2122

[bib21] Record M, Carayon K, Poirot M, Silvente-Poirot S. Exosomes as new vesicular lipid transporters involved in cell-cell communication and various pathophysiologies. Biochim Biophys Acta 2014; 1841: 108–120. 24140720 10.1016/j.bbalip.2013.10.004

[bib22] Ibrahim AG, Cheng K, Marban E. Exosomes as critical agents of cardiac regeneration triggered by cell therapy. Stem Cell Rep 2014; 2: 606–619. 10.1016/j.stemcr.2014.04.006 PMC4050492 24936449

[bib23] Lee HJ, Cho ED, Lee KW, Kim JH, Cho SG, Lee SJ. Autophagic failure promotes the exocytosis and intercellular transfer of alpha-synuclein. Exp Mol Med 2013; 45: e22. 23661100 10.1038/emm.2013.45 PMC3674407

[bib24] Feeney EJ, Spampanato C, Puertollano R, Ballabio A, Parenti G, Raben N. What else is in store for autophagy? Exocytosis of autolysosomes as a mechanism of TFEB-mediated cellular clearance in Pompe disease. Autophagy 2013; 9: 1117–1118. 23669057 10.4161/auto.24920 PMC3722326

[bib25] Kim J, Kundu M, Viollet B, Guan KL. AMPK and mTOR regulate autophagy through direct phosphorylation of Ulk1. Nat Cell Biol 2011; 13: 132–141. 21258367 10.1038/ncb2152 PMC3987946

[bib26] Yamamoto A, Tagawa Y, Yoshimori T, Moriyama Y, Masaki R, Tashiro Y. Bafilomycin A1 prevents maturation of autophagic vacuoles by inhibiting fusion between autophagosomes and lysosomes in rat hepatoma cell line, H-4-II-E cells. Cell Struct Funct 1998; 23: 33–42. 9639028 10.1247/csf.23.33

[bib27] Listenberger LL, Ory DS, Schaffer JE. Palmitate-induced apoptosis can occur through a ceramide-independent pathway. J Biol Chem 2001; 276: 14890–14895. 11278654 10.1074/jbc.M010286200

[bib28] Bligh EG, Dyer WJ. A rapid method of total lipid extraction and purification. Can J Biochem Physiol 1959; 37: 911–917. 13671378 10.1139/o59-099

[bib29] Rodriguez-Sureda V, Peinado-Onsurbe J. A procedure for measuring triacylglyceride and cholesterol content using a small amount of tissue. Anal Biochem 2005; 343: 277–282. 15993372 10.1016/j.ab.2005.05.009

[bib30] Klionsky DJ, Abdalla FC, Abeliovich H, Abraham RT, Acevedo-Arozena A, Adeli K et al. Guidelines for the use and interpretation of assays for monitoring autophagy. Autophagy 2012; 8: 445–544. 22966490 10.4161/auto.19496 PMC3404883

